# Phosphodiester content measured in human liver by in vivo ^31^P MR spectroscopy at 7 tesla

**DOI:** 10.1002/mrm.26635

**Published:** 2017-02-28

**Authors:** Lucian A.B. Purvis, William T. Clarke, Ladislav Valkovič, Christina Levick, Michael Pavlides, Eleanor Barnes, Jeremy F. Cobbold, Matthew D. Robson, Christopher T. Rodgers

**Affiliations:** ^1^ Oxford Centre for Clinical Magnetic Resonance Research (OCMR) University of Oxford, Level 0, John Radcliffe Hospital Oxford United Kingdom; ^2^ Department of Imaging Methods Institute of Measurement Science, Slovak Academy of Sciences Bratislava Slovakia; ^3^ Translational Gastroenterology Unit University of Oxford United Kingdom.

**Keywords:** ^31^P, phosphorus, magnetic resonance spectroscopy, human, in vivo, liver, cirrhosis, 7 Tesla

## Abstract

**Purpose:**

Phosphorus (^31^P) metabolites are emerging liver disease biomarkers. Of particular interest are phosphomonoester and phosphodiester (PDE) “peaks” that comprise multiple overlapping resonances in ^31^P spectra. This study investigates the effect of improved spectral resolution at 7 Tesla (T) on quantifying hepatic metabolites in cirrhosis.

**Methods:**

Five volunteers were scanned to determine metabolite T_1_s. Ten volunteers and 11 patients with liver cirrhosis were scanned at 7T. Liver spectra were acquired in 28 min using a 16‐channel ^31^P array and 3D chemical shift imaging. Concentrations were calculated using γ‐adenosine‐triphosphate (γ‐ATP) = 2.65 mmol/L wet tissue.

**Results:**

T_1_ means ± standard deviations: phosphatidylcholine 1.05 ± 0.28 s, nicotinamide‐adenine‐dinucleotide (NAD^+^) 2.0 ± 1.0 s, uridine‐diphosphoglucose (UDPG) 3.3 ± 1.4 s. Concentrations in healthy volunteers: α‐ATP 2.74 ± 0.11 mmol/L wet tissue, inorganic phosphate 2.23 ± 0.20 mmol/L wet tissue, glycerophosphocholine 2.34 ± 0.46 mmol/L wet tissue, glycerophosphoethanolamine 1.50 ± 0.28 mmol/L wet tissue, phosphocholine 1.06 ± 0.16 mmol/L wet tissue, phosphoethanolamine 0.77 ± 0.14 mmol/L wet tissue, NAD^+^ 2.37 ± 0.14 mmol/L wet tissue, UDPG 2.00 ± 0.22 mmol/L wet tissue, phosphatidylcholine 1.38 ± 0.31 mmol/L wet tissue. Inorganic phosphate and phosphatidylcholine concentrations were significantly lower in patients; glycerophosphoethanolamine concentrations were significantly higher (*P* < 0.05).

**Conclusion:**

We report human in vivo hepatic T_1_s for phosphatidylcholine, NAD^+^, and UDPG for the first time at 7T. Our protocol allows high signal‐to‐noise, repeatable measurement of metabolite concentrations in human liver. The splitting of PDE into its constituent peaks at 7T may allow more insight into changes in metabolism. Magn Reson Med 78:2095–2105, 2017. © 2017 The Authors Magnetic Resonance in Medicine published by Wiley Periodicals, Inc. on behalf of International Society for Magnetic Resonance in Medicine. This is an open access article under the terms of the Creative Commons Attribution License, which permits use, distribution and reproduction in any medium, provided the original work is properly cited.

## INTRODUCTION

Liver disease is becoming increasingly prevalent 1, now estimated to affect more than 10% of adults in the Europe and America 2. Liver biopsy is the accepted standard test for diagnosis, but biopsy is invasive, can cause pain and bleeding, and has nonnegligible associated mortality 3. In addition, because liver biopsies sample less than 0.0002% of the liver by mass 4, biopsy suffers undesirable sampling variability 5, 6, 7. This variability can be seen in studies of paired biopsies for chronic hepatitis C 8, 9, and has been blamed for causing “substantial misdiagnosis and staging inaccuracy” in nonalcoholic fatty liver disease (NAFLD) 10.

Phosphorus‐31 (^31^P) metabolites measured using in vivo magnetic resonance spectroscopy (MRS) are emerging biomarkers for several liver diseases including NAFLD 11, 12, 13 and cirrhosis 14. The phosphomonoester (PME) and phosphodiester (PDE) peaks of the ^31^P liver spectrum are particularly promising 15, 16. The PME “peak” is believed to contain contributions from phosphocholine (PC) and phosphoethanolamine (PE), which are cell membrane precursors 17. The PDE peak comprises signal from glycerophosphocholine (GPC), and glycerophosphoenthanolamine (GPE), cell membrane degradation products 17. Close to the PDE resonance, there is an overlap of signals from phosphoenolpyruvate (PEP) and, more importantly, from phosphatidylcholine (PtdC), which is a predominant phospholipid component of bile 18, 19, 20. At lower field strengths, the spectral resolution needed to individually resolve these five peaks is only possible with ^1^H decoupling 21, 22. This is no longer required at 7T, because the frequency dispersion of peaks increases proportionally to B_0_ 18, 23. Resolution of individual metabolites can allow more metabolite changes to be visible. For example, PC and PE have been reported at 7T to have opposing changes in human breast cancer, which is not visible in a combined PME peak 24. In the PDE region, there is also a broad baseline resonance from the endoplasmic reticulum 25. In vitro, this resonance broadens from 210 Hz at 1.9 T to 7000 Hz at 7T and shifts toward lower ppm 25. This broadening was observed in vivo in rats at 8.5T 26. We expect these two effects to combine with the inherent increase in the signal‐to‐noise ratio (SNR) for ^31^P‐MRS at 7T 27 to allow more accurate quantitation of the PME and PDE peaks.

To quantitate these peaks we need to correct for the saturation effects in our CSI protocol and T_1_ values must be known. Although there are literature values for most MR‐visible ^31^P liver metabolites at 7T, they do not include PEP/PtdC, nicotinamide adenine dinucleotide (NAD^+^), or uridine diphosophoglucose (UDPG). To properly investigate the PDE region, at least the PEP/PtdC T_1_ must be known. Therefore, the liver metabolite T_1_ values were determined first. The data quality of the normal protocol, including inherent error and repeatability, was then assessed. Finally this protocol was used for quantitation of the PDE region in volunteers and in patients with cirrhotic livers.

## METHODS

Patient recruitment to the study was granted ethical approval from the UK National Research Ethics Service (13/SC/0243) and was conducted according to the principles of the 1975 Declaration of Helsinki. All subjects were scanned supine after an overnight fast using an actively shielded whole‐body Magnetom 7T (Siemens, Erlangen, Germany).

Transverse, sagittal and coronal ^1^H fast low angle shot (FLASH) stacks of images for localization, and dual‐echo field maps for B_0_ shimming were acquired using a 10 cm ^1^H transmit/receive (Tx/Rx) loop coil (Rapid Biomedical, Germany). B_0_ shims were optimized over a region of interest (ROI) covering the entire liver 28. The ^1^H coil was then replaced with a 16‐channel ^31^P receive array coil (Rapid Biomedical, Germany), consisting of a single 28 × 27 cm^2^ transmit loop and a 4 × 4 matrix of 8 × 5.5 cm^2^ diameter flexible receive loops 29. Signal from a spherical phenylphosphonic acid (PPA) fiducial, in housing on the rear of the coil, was acquired using a series of nonlocalized inversion‐recovery free induction decays (FIDs) and used to calculate transmit efficiency. Three orthogonal ^31^P FLASH images were then acquired to localize the fiducial, and used to calculate the coil position 27.

**Figure 1:**
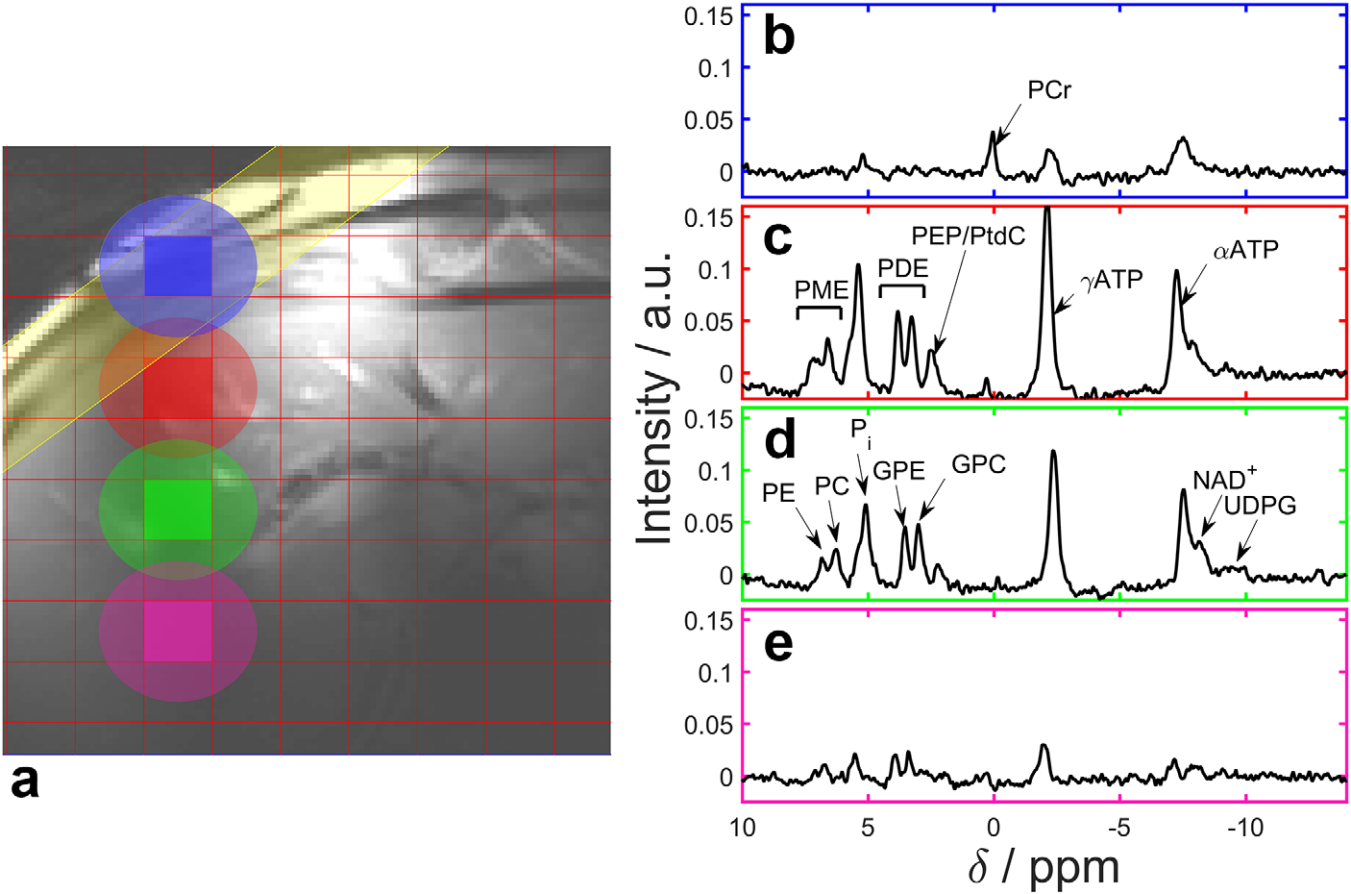
Typical liver ^31^P‐MRS spectra acquired at 7T in the right lobe of the liver using our 3D UTE‐CSI protocol. **a**: The ^1^H FLASH localizer. The red lines mark the CSI grid, the yellow band marks the outer volume BISTRO suppression. The spectrum for each colored voxel is given on the right side. The ellipses show the extent of the full width at half maximum of the point spread function for each voxel. **b**: A suppressed muscle spectrum taken from the blue voxel. **c–e**: Liver spectra from the red, green and pink voxels, respectively, showing the change as they are taken from deeper in the liver.

In both T_1_ determination and normal protocol, the excitation pulse (a 0.5 ms hard pulse preceded by a numerically optimized portion to flatten the frequency response in a total 2.4 ms duration, described in Rodgers et al) 27 gave a uniform 2000 Hz bandwidth covering all metabolites from UDPG to PC (‐9.5 to 7 ppm) 30. The signal was localized using an acquisition‐weighted 3D ultrashort echo time chemical shift imaging (UTE‐CSI) sequence 31, which uses the shortest possible echo time at each point of k‐space. In each case, a B_1_‐insensitive train to obliterate signal (BISTRO) saturation band was used to suppress overlying skeletal muscle 32 (see Fig. 1). No ^1^H‐decoupling or nuclear Overhauser effect (NOE) enhancement was used.

### T_1_ Determination

Five male volunteers (28 ± 6 years, body mass index (BMI) 21.3 ± 2.9 kg.m^‐2^) were scanned to determine metabolite T_1_ values. A Look‐Locker chemical shift imaging (LL‐CSI) inversion recovery pulse sequence with 24 effective inversion times (TIs) between 50 ms and 15.55 s was used as previously described 27. Signals were localized to a 6 × 6 × 4 acquisition weighted matrix (interpolated to 8 × 8 × 8 by zero‐padding of k‐space before analysis) and a 200 × 240 × 270 mm^3^ field of view. The LL‐CSI sequence was run twice in each subject, first with inversion at +666 Hz [relative to skeletal phosphocreatine (PCr)] and second at ‐584 Hz (relative to PCr). The inversion pulse bandwidth of 1020 Hz ensured that every metabolite of interest was inverted in at least one data set. One voxel that was fully localized to the liver was manually selected per subject. The following criteria were considered: resolution of the PtdC, PDE and PME regions, skeletal muscle contamination and SNR. Data were fitted to a Bloch simulation of the pulse sequence, as described in the Supporting Information, and the fitting steps are given in Supporting Table S1.

### Acquisition

Ten volunteers (six male and four female, 27 ± 5 years; BMI 22.5 ± 1.5 kg.m^‐2^) and eleven patients with cirrhosis of the liver (seven male and four female, 61 ± 6 years; BMI 29.5 ± 6.9 kg.m^‐2^) were scanned. The patients were recruited based on cirrhosis established using clinical, biochemical or radiological criteria. Five patients had been previously diagnosed with hepatitis C, three with nonalcoholic fatty liver disease, two with alcoholic steatohepatitis, and one with autoimmune hepatitis.

A 1 s T_R_, 10 average acquisition‐weighted UTE‐CSI sequence was used to acquire a 16 × 16 × 8 matrix of liver spectra over a 270 × 240 × 200 mm^3^ field of view in a total acquisition duration of 28 min. To test the repeatability of the protocol, each healthy volunteer was measured a second time in the same session, after full repositioning and reshimming.

### Processing

On the scanner, spectra from each receive element were combined with whitened singular value decomposition (WSVD) combination 33, 34 to give a single uniform noise spectrum for each voxel.

**Figure 2:**
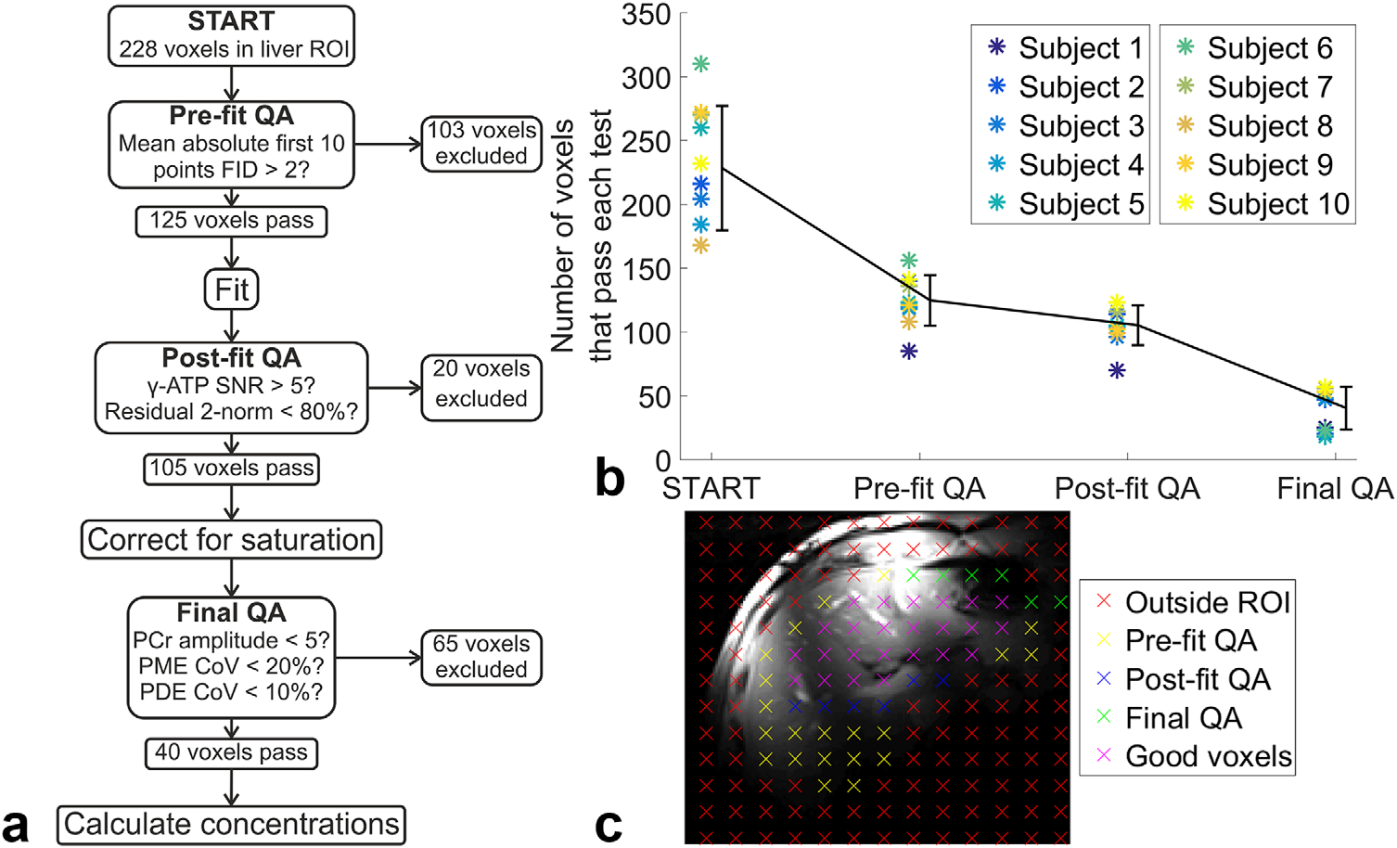
**a**: The decision tree of quality assurance (QA) tests for excluding voxels in the analysis of the CSI grid of the liver. The mean number of voxels remaining at each step are given. **b**: The number of voxels that pass each test. The black line shows the mean ± SD of the values. The stars show individual values, with each color representing a different subject. **c**: The locations of a single slice of CSI voxels overlaid on a ^1^H localizer. Each color indicates a different exclusion parameter.

The full grid of CSI spectra was then loaded into Matlab (MathWorks, Natick, MA). A ROI was manually drawn around the liver on the transverse ^1^H localizer. Voxels were excluded if they were outside the ROI or if the mean of the absolute of the first 10 points of the FID was less than 2.0 (the noise level in each combined spectrum is scaled by WSVD to be 1/√2) 33. Each voxel was fitted using a linewidth‐constrained, open‐source, Matlab spectroscopy fitting tool 35. The additional linewidths were calculated using five 3D CSI datasets acquired from healthy volunteers. The prior knowledge used in the fitting may be found in the Supporting Table S2. After fitting, γ‐ATP SNR was calculated as the maximum intensity signal over noise standard deviation (SD) from the spectrum after it had been apodized to match the γ‐ATP linewidth. Voxels with γ‐ATP SNR less than five, or residual 2‐norm greater than 80% of the original were excluded. Saturation correction factors were calculated using the measured T_1_ values, accounting for actual flip angle in each voxel calculated from the per‐subject B_1_ maps computed using Biot‐Savart's law (see Supporting Fig. S1, which is available online, for phantom validation) and adjusted for coil loading and position using the PPA FIDs and FLASH images 27. Voxels were discarded if they had PCr amplitude greater than five (i.e. contaminated by skeletal muscle), or a Cramér‐Rao lower bound (CRLB), i.e., a measure of the uncertainty in the fit 36, greater than 20% for any PME peak or greater than 10% for any PDE peak. Histograms were used to select the skeletal PCr amplitudes below the normal distribution and PME or PDE CRLB more than two SD above the mean. Any voxels remaining after this automatic quality assurance procedure are referred to below as “high quality”. Figure 2a shows the decision tree for exclusion.

Histograms of the data before and after exclusion were checked manually to ensure that no bias was introduced by using percentage CRLB as exclusion criteria 37.

Concentrations were calculated using γ‐ATP as an endogenous reference by assuming that the filling fraction and sensitivities of γ‐ATP and each of the other metabolites were equal, and applying Equation [4] from Bottomley 38. The concentration of γ‐ATP was taken as 2.65 mmol/L wet tissue based on a 2.51 ± 0.61 mmol/kg wet weight 39, and 1.054 kg/L specific gravity of the liver 40. Metabolites were also quantified as a ratio to the total phosphorus (i.e., the sum of all metabolite peaks). The mean and SD for each subject was calculated from the high quality voxels. The within‐subject SDs were calculated using the SDs between the mean values for the repeated volunteer scans, and the coefficients of repeatability (CoRs) were calculated as 1.96 × √2 × the within‐subject SD. The mean absolute difference between two repeated scans should be less than the CoR in 95% of cases. For comparison with the CoRs for ^31^P‐MRS from three voxels in the heart at 3T 30, the CoRs were also calculated using the average values from three randomly selected voxels rather than all the available voxels. This was repeated 100 times and the average CoR was used. Bland‐Altman plots were used to further analyze the repeatability of the protocol. Welch's t‐test was used to compare means, allowing for unequal variances 41. An F‐test was used to compare variances.

To calculate the true volume of the CSI voxels, full width at half maximum of the theoretical point spread function (PSF) 42, 43 was used in each dimension to form an ellipsoid for each voxel. When several voxels were used, care was taken to count the contribution from overlapping volumes only once.

The liver volume of each subject in mL was approximated by using Equation [1] (from Du Bois and Du Bois) 44 and Equation [2] (from Heinemann et al) 45. This volume was then used to calculate the coverage achieved in this study.

(1) $$\text{Body}\;\text{surface}\;\text{area}\;\left(\text{BSA}\right)\;/\;m^{2}=0.007184\;\times {\left(\text{mass}\;/\;\text{kg}\;\right)}^{0.425}\;\times \left(\text{height}\;/\;m\right){\;}^{0.725}\;$$

(2) $$\text{Liver}\;\text{volume}\;/\;\text{mL}\;=1072.8\;\times \left(\text{BSA}\;/{\;m}^{2}\right)\;-347.5\;$$

## RESULTS

Figure 1 shows a typical liver spectrum acquired from a volunteer with our standard protocol as described above. The individual PME and PDE peaks are clearly separated. The PEP/PtdC peak is distinct from the GPC peak. The NAD^+^ peak is visible on the side of the αATP peak, and there is a small UDPG peak.

**Figure 3:**
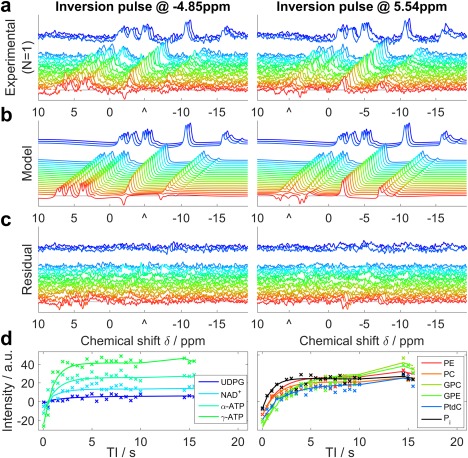
Illustration of ^31^P Look‐Locker CSI fitting for a typical set of liver data. **a**: Raw spectra from a single voxel of a single subject. Each line shows a different TI, with a gap to indicate the break for magnetization recovery in the pulse sequence. **b**: The model spectra that were fitted simultaneously to the experimental data. **c**: The residual error after fitting. a–c: Images are plotted with the same scaling. “^” marks the central frequency of the inversion pulse. **d**: The absolute intensity sampled at the fitted frequency of each metabolite. Each “x” marks an experimental TI, and the lines show the simulated data. These panels are drawn to help interpret the spectra in a–c, but were not used in the T_1_ analysis.

An illustration of the T_1_ fitting method is given in Figure 3. Healthy human liver metabolite T_1_ values are reported and compared with published values in Table 1. The full width half maximum PSF size of a single LL‐CSI protocol voxel was 377 mL. The newly determined T_1_ means ± SDs were: PtdC 1.05 ± 0.28 s, NAD^+^ 2.0 ± 1.0 s, UDPG 3.3 ± 1.4 s. The T_1_ for P_i_ was 1.34 ± 0.15 s, GPC was 3.9 ± 1.3 s, and GPE was 4.4 ± 1.1 s.

**Table 1:** T_1_ Values from Each Subject Are Given with Cramer‐Rao Lower Bounds of the Fit^a^

Metabolite	Subject 1	Subject 2	Subject 3	Subject 4	Subject 5	Mean ± SD	Literature values
α‐ATP / s	0.66 ± 0.04	0.42 ± 0.04	0.55 ± 0.07	0.62 ± 0.09	0.55 ± 0.05	0.56 ± 0.09	0.46 ± 0.07^b^
γ‐ATP / s	0.52 ± 0.03	0.82 ± 0.03	0.62 ± 0.04	0.46 ± 0.04	0.44 ± 0.03	0.57 ± 0.15	0.50 ± 0.08^b^
P_i_ / s	1.32 ± 0.07	1.40 ± 0.06	1.11 ± 0.09	1.51 ± 0.14	1.34 ± 0.10	1.34 ± 0.15	0.70 ± 0.33^b^
GPC / s	3.5 ± 0.4	3.8 ± 0.3	6.0 ± 3.6	3.7 ± 0.3	2.5 ± 0.3	3.9 ± 1.3	5.94 ± 0.73^b^
GPE / s	4.4 ± 0.3	4.0 ± 0.3	6.2 ± 1.9	3.4 ± 0.4	3.9 ± 0.3	4.4 ± 1.1	6.19 ± 0.91^b^
PC / s	1.5 ± 0.3	2.0 ± 0.2	1.8 ± 0.3	3.1 ± 0.6	2.9 ± 0.7	2.3 ± 0.7	3.74 ± 1.31^b^
PE / s	3.4 ± 0.3	3.9 ± 0.5	4.4 ± 1.0	5.4 ± 0.5	2.4 ± 0.3	3.9 ± 1.1	4.41 ± 1.55^b^
PtdC/ s	1.37 ± 0.25	1.09 ± 0.04	0.70 ± 0.04	1.25 ± 0.17	0.86 ± 0.03	1.05 ± 0.28	0.97 ± 0.30^c^
NAD^+^ / s	1.3 ± 0.2	1.6 ± 0.2	1.0 ± 0.3	3.1 ± 0.5	3.2 ± 0.4	2.0 ± 1.0	2.0 ± 1.0^d^
UDPG / s	1.9 ± 0.8	1.9 ± 0.6	3.7 ± 1.3	5.0 ± 1.7	4.0 ± 1.4	3.3 ± 1.4	‐

a The mean and literature values are given with SD.

b 7T values from Chmelík et al. 23.

c 3T value from Bierwagen et al. 20.

d 1.5T value from Li et al. 21.

In the main acquisition, an average of 228 ± 49 voxels fell within the ROI, which had a coverage of 92 ± 34%, i.e., approximately the whole liver. 125 ± 20 voxels passed the prefit quality assurance test and 105 ± 16 had a good fit and γ‐ATP SNR greater than 5. This left 40 ± 17 high quality voxels, which had good SNR, were well fitted, and were reliably localized to the liver (see Fig. 2).

The nominal voxel size was 6.3 mL, but acquisition weighting means that the 50% maximum point‐spread‐function volume was 47.1 mL 42, 43. The average liver volume was 1.70 ± 0.20 L, and accounting for voxel overlap the average volume within each liver that was analyzed was 0.77 ± 0.17 L. The total liver coverage was, therefore, 46 ± 13%. The mean γ‐ATP linewidth is 48 ± 13 Hz, and the average spectral γ‐ATP SNR (amplitude / noise SD) was 22 ± 2. The average ratio of the residual PCr signal to ATP was 0.24 ± 0.04 in the liver.

Metabolite concentrations computed for both sets of volunteer scans are given in Table 2. The total PDE concentration measured in healthy volunteers was 3.84 ± 0.68 mmol/L wet tissue, of which 2.34 ± 0.46 mmol/L wet tissue was from GPC and 1.50 ± 0.28 mmol/L wet tissue from GPE. The PtdC/PEP concentration was 1.38 ± 0.31 mmol/L wet tissue.

**Table 2:** Mean ± SD of Concentrations for 10 Normal Volunteers and 11 Patients with Cirrhotic Livers from a 28‐min 3D CSI Acquisition^a^

Metabolite	Normal liver: Scan 1 / mmol/L wet tissue	Normal liver : Scan 2 / mmol/L wet tissue	Cirrhotic liver / mmol/L wet tissue	Difference between normal (scan 1) and cirrhotic livers/ %	*P*‐Value
α‐ATP	2.74 ± 0.11	2.69 ± 0.18	2.81 ± 0.11	2.3	0.10
P_i_	2.23 ± 0.20	2.18 ± 0.18	1.96 ± 0.23	−12.2	0.005**
GPC	2.34 ± 0.46	2.35 ± 0.48	2.55 ± 0.59	9.1	0.18
GPE	1.50 ± 0.28	1.49 ± 0.21	1.82 ± 0.47	20.9	0.04*
PC	1.06 ± 0.16	0.99 ± 0.17	0.93 ± 0.24	−12.2	0.08
PE	0.77 ± 0.14	0.70 ± 0.11	0.76 ± 0.11	−1.47	0.42
PtdC/PEP	1.38 ± 0.31	1.35 ± 0.38	1.06 ± 0.32	−23.2	0.016*
NAD^+^	2.37 ± 0.14	2.33 ± 0.27	2.41 ± 0.38	1.7	0.38
UDPG	2.00 ± 0.22	2.06 ± 0.17	1.99 ± 0.37	−0.6	0.47
PDE	3.84 ± 0.68	3.84 ± 0.66	4.37 ± 0.92	13.7	0.08
PME	1.83 ± 0.27	1.69 ± 0.24	1.69 ± 0.28	−7.7	0.12

a The concentrations are based on a 2.65 mmol/L wet tissue γ‐ATP concentration. This assumption is reasonable for the normal volunteers, but does not necessarily hold for cirrhosis. The PDE concentration is the sum of the GPC and GPE concentrations, and the PME concentration is the sum of the PC and PE concentrations. Significant differences between healthy and cirrhotic concentrations are marked with stars: * *P* < 0.05, ** *P* < 0.01.

**Figure 4:**
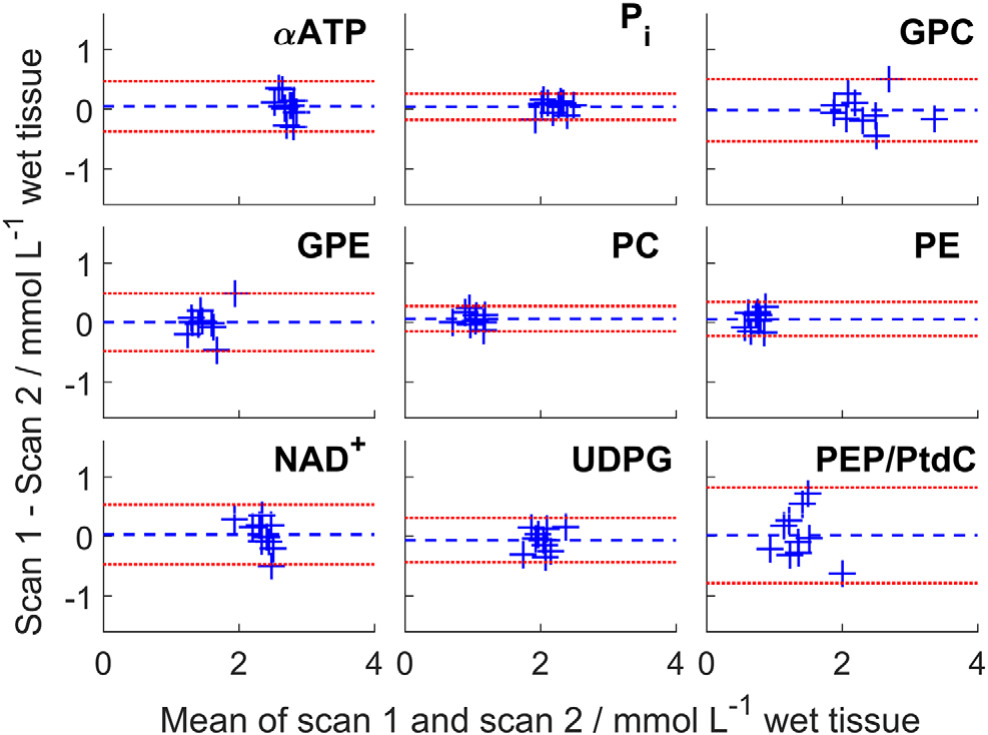
Bland‐Altman plots of the concentrations of the two repeatability scans. Each blue cross marks a different subject. The blue dashed line is the mean difference and the red dashed line is 1.96 × SD, i.e., the 95% confidence interval.

The Bland‐Altman plot for the repeatability scans is shown in Figure 4. The average difference between the two measurements was not significant (*P* < 0.1 for all peaks). The CoRs, CRLB, and inter‐ and intrasubject SD are given in Table 3. The average γ‐ATP CRLB was 1.6%.The PtdC/PEP peak has the largest absolute CoR (55.5%) and the next largest was PE (38.0%). Each of the three‐voxel CoRs are significantly larger than the full CoRs (*P* < 0.05 for all peaks).

**Table 3:** Coefficients of Repeatability (CoR) in Normals Are Given ± 95% Confidence Intervals^a^

Metabolite	CoR / mmol/L wet tissue	3‐Voxel CoR / mmol/L wet tissue	Mean CRLB / mmol/L wet tissue	Mean intrasubject SD (CoV) / mmol/L wet tissue	Intersubject SD (CoV) / mmol/L wet tissue
α‐ATP	0.41 (15.1%) ± 0.23	0.83 (30.4%) ± 0.61	0.0269 (1.1%) ± 0.0036	0.45 (16.4%) ± 0.13	0.11 (3.9%)
P_i_	0.23 (10.1%) ± 0.08	0.68 (30.3%) ± 0.46	0.0516 (2.3%) ± 0.0047	0.40 (17.9%) ± 0.09	0.20 (8.9%)
GPC	0.50 (21.2%) ± 0.35	1.28 (54.8%) ± 0.88	0.0500 (2.3%) ± 0.0039	0.75 (31.9%) ± 0.17	0.46 (19.5%)
GPE	0.46 (30.5%) ± 0.39	0.84 (55.9%) ± 0.58	0.0386 (2.7%) ± 0.0036	0.47 (31.5%) ± 0.12	0.28 (18.5%)
PC	0.24 (22.9%) ± 0.16	0.74 (69.9%) ± 0.54	0.0431 (4.5%) ± 0.0039	0.46 (43.8%) ± 0.17	0.16 (14.7%)
PE	0.30 (38.9%) ± 0.14	0.65 (84.3%) ± 0.47	0.0376 (5.5%) ± 0.0041	0.37 (48.0%) ± 0.12	0.14 (17.8%)
PtdC	0.76 (55.5%) ± 0.45	1.85 (134.5%) ± 1.59	0.0694 (7.1%) ± 0.0066	0.70 (29.7%) ± 0.14	0.31 (22.7%)
NAD^+^	0.48 (20.4%) ± 0.30	1.20 (50.6%) ± 0.80	0.0654 (3.4%) ± 0.0069	0.78 (38.8%) ± 0.14	0.14 (6.0%)
UDPG	0.37 (18.5%) ± 0.20	1.29 (64.6%) ± 0.85	0.0610 (3.8%) ± 0.0063	1.07 (77.8%) ± 0.47	0.22 (10.9%)

a The concentrations are based on a 2.65 mmol/L wet tissue γ‐ATP concentration, with percentage of peak concentration given in brackets. The intrasubject SDs are reduced compared to fully independent measurements, as there is no correction for voxel overlap.

Average metabolite concentrations for cirrhotic liver are also given in Table 2, and the concentrations for individual patients are given in Table 4 with their diagnoses. The mean total liver volume for cirrhotic liver was 1.76 ± 0.21 L, and the mean volume analyzed in cirrhotic liver was 0.78 ± 0.34 L from 42 ± 23 voxels. The coverage was 46 ± 21%. The average γ‐ATP linewidth was 44 ± 19 Hz for patients, and the SNR was 20 ± 6. These are not significantly different from in healthy volunteers. The lowest coverages were 18% for a patient with a BMI of 43.9 kg.m^‐2^, and 14.5% for a patient with BMI of 35.6 kg.m^‐2^.

**Table 4:** Metabolite Concentrations of 11 Patients with Liver Cirrhosis (Mean ± Intervoxel SD)^a^

Subject	1	2	3	4	5	6	7	8	9	10	11
Diagnosis	HCV	HCV	HCV	HCV	HCV	NAFLD	NAFLD	NAFLD	ASH	ASH	AIH
α‐ATP / mmol/L wet tissue	3.04 ± 0.37	2.84 ± 0.24	2.74 ± 0.29	2.69 ± 0.30	2.67 ± 0.31	2.81 ± 0.33	2.92 ± 0.34	2.76 ± 0.31	2.71 ± 0.58	2.77 ± 0.45	2.89 ± 0.40
P_i_ / mmol/L wet tissue	2.43 ± 0.42	2.14 ± 0.23	1.91 ± 0.31	1.88 ± 0.26	2.09 ± 0.52	1.76 ± 0.22	1.80 ± 0.27	1.67 ± 0.28	1.78 ± 0.22	2.22 ± 0.14	1.85 ± 0.32
GPC / mmol/L wet tissue	1.92 ± 0.64	1.47 ± 0.45	2.78 ± 0.87	2.78 ± 0.71	2.53 ± 1.05	2.09 ± 0.40	2.57 ± 0.70	3.70 ± 1.02	2.93 ± 0.37	2.40 ± 0.41	2.88 ± 0.82
GPE / mmol/L wet tissue	1.94 ± 0.65	0.87 ± 0.29	2.46 ± 0.66	1.57 ± 0.41	1.62 ± 0.59	1.49 ± 0.27	1.55 ± 0.39	1.94 ± 0.60	2.09 ± 0.33	2.48 ± 0.29	1.99 ± 0.58
PC / mmol/L wet tissue	1.18 ± 0.37	1.40 ± 0.41	0.91 ± 0.32	1.21 ± 0.33	0.91 ± 0.51	0.75 ± 0.26	0.82 ± 0.25	0.71 ± 0.24	0.75 ± 0.16	0.94 ± 0.26	0.66 ± 0.27
PE / mmol/L wet tissue	0.83 ± 0.39	0.82 ± 0.35	0.81 ± 0.28	0.59 ± 0.26	0.77 ± 0.34	0.90 ± 0.24	0.59 ± 0.20	0.67 ± 0.31	0.73 ± 0.26	0.92 ± 0.30	0.68 ± 0.36
NAD^+^ / mmol/L wet tissue	2.33 ± 0.60	2.49 ± 0.66	2.12 ± 0.65	2.64 ± 0.53	1.98 ± 0.45	3.07 ± 0.49	1.92 ± 0.57	2.40 ± 0.66	2.17 ± 0.50	2.88 ± 0.49	2.55 ± 0.69
UDPG / mmol/L wet tissue	2.15 ± 0.77	1.69 ± 0.69	2.00 ± 0.78	2.44 ± 1.10	1.74 ± 0.73	2.43 ± 0.74	1.53 ± 0.77	2.37 ± 1.00	2.07 ± 0.89	1.37 ± 0.58	2.08 ± 0.91
PEP/PtdC / mmol/L wet tissue	1.37 ± 0.87	1.39 ± 0.91	0.81 ± 0.22	1.35 ± 1.14	1.16 ± 0.58	0.61 ± 0.19	1.37 ± 1.19	0.87 ± 0.39	0.63 ± 0.26	0.79 ± 0.27	1.27 ± 1.38
PDE / mmol/L wet tissue	3.86 ± 1.21	2.35 ± 0.71	5.25 ± 1.49	4.35 ± 1.09	4.14 ± 1.59	3.58 ± 0.65	4.11 ± 1.07	5.65 ± 1.58	5.03 ± 0.68	4.88 ± 0.63	4.87 ± 1.36
PME / mmol/L wet tissue	2.00 ± 0.68	2.21 ± 0.64	1.72 ± 0.55	1.80 ± 0.47	1.67 ± 0.68	1.66 ± 0.33	1.42 ± 0.36	1.38 ± 0.49	1.49 ± 0.25	1.86 ± 0.33	1.34 ± 0.52

a Five have been diagnosed with hepatitis C (HCV), three with NAFLD, two with alcoholic steatohepatitis (ASH), and one with auto‐immune hepatitis (AIH). The intervoxel SDs are reduced compared to fully independent measurements, as there is no correction for voxel overlap. Intersubject means are given in Table 2.

**Figure 5:**
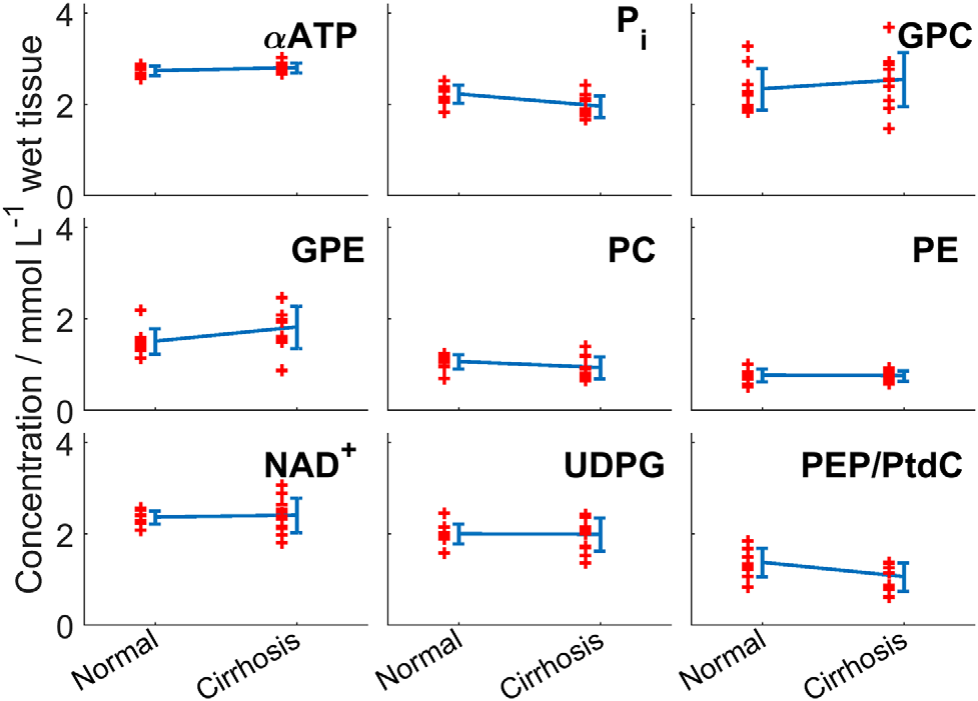
A scatter plot of the concentrations of nine different peaks in normal volunteers and patients. Each red “+” marks a subject. The blue line shows the mean ± SD of each group. P_i_ and PtdC/PEP are significantly lower in cirrhosis than in normal volunteers, and GPE is significantly higher (*P* < 0.05).

Figure 5 shows the concentrations for each volunteer and patient. There are significant reduction in P_i_ (12%; *P* = 0.005), and PEP/PtdC concentrations (23%; *P* = 0.016), and a trend to a reduction in PC (12%; *P* = 0.08). There was a significant increase in GPE concentrations (21%; *P* = 0.04). Only NAD^+^ has a significantly larger variance in patients than in healthy volunteers (F‐test; *P* = 0.003). These differences were also significant when comparing the metabolite ratios to total phosphorus (shown in Supporting Table S3).

## DISCUSSION

This study reports T_1_ values for PtdC/PEP, NAD^+^, and UDPG, which have not previously been measured, as well as the other metabolites visible in ^31^P spectra. Our main protocol, using CSI localization and a receive array, gave 46 ± 21% coverage of the liver, while retaining spectra localized to 47.1 mL, which allowed regions of the liver contaminated, e.g., by bile to be excluded. The high average γ‐ATP SNR of 20 ± 4 led to low errors in quantitation (γ‐ATP CRLB of 1.6%) and there is good spectral resolution of the PME and PDE regions. We have established the error and repeatability in our protocol in ten volunteers. And we have shown that our protocol remains effective in cirrhosis patients, in whom we saw a significant difference in P_i,_ PEP/PtdC, and GPE compared with volunteers.

### T_1_ Determination

The 95% confidence intervals of our T_1_ values and those in the literature overlapped for all metabolites except for P_i_, GPE, and GPC 23. The limited bandwidth of our inversion pulse meant that two inversions were required. Magnetization transfer could, therefore, have reduced the apparent T_1_ of the P_i_ or ATP peaks. However, as the T_1_ values were longer than those of Chmelík et al, this effect does not appear to be significant. Chmelík et al performed two IR experiments, one optimized for short T_1_ metabolites (eight TI values between 0.02 and 2 s) and the other for long T_1_ metabolites (eight TI values between 0.1 and 20 s). The effective TI values in our study (24 between 0.05 s and 15.55 s) are more optimally spaced for intermediate T_1_ metabolites. This could be the cause of the differences seen in metabolite T_1_ values. Valkovič et al used eight TI values between 0.08 and 3 s, and reported an apparent T_1_ of P_i_ with saturated γ‐ATP of 0.77 ± 0.16 s 46. This is consistent with our result (P_i_ T_1_ = 1.34 ± 0.15), as the true T_1_ would be longer than the apparent T_1_ under saturation.

The three new metabolite T_1_ values that we have measured all fall in the intermediate range, and our protocol should, therefore, give an accurate result. Furthermore, previously reported T_1_ relaxation times at 1.5T have shown shorter values for ATP, P_i_, PME, and PDE in human liver compared with those in calf muscle 47, and a similar effect can be seen at 3T 48, 49. Extrapolating to 7T, it could be expected that the average T_1_ values for PDE should be shorter than 5.7 ± 1.5 s 49, which is consistent with our findings. Similarly, the T_1_ of ATP should be shorter than 1.8 ± 0.1 s 49, and closer to the values seen in the liver at lower field strength (0.4–0.9 s) 21, 47, 48. It is perhaps surprising that T_1_ values in the liver differ so much from those in skeletal muscle and prostate at 7T. Further study will be required to determine the origins of this difference.

### Data Quality

Quality assurance in postprocessing is important for CSI methods because the matrix of voxels acquired typically vary widely in SNR, B_o_ homogeneity and contamination. Automatic methods for the selection of high quality voxels reduce observer bias and the need for postprocessing by experts, and reduce the time required for quality assurance, which is necessary for a protocol to be clinically viable. The trade‐off is the possibility of including voxels that would be discarded by an expert. In this study, we checked the performance of the quality assurance pipeline shown in Figure 2 by manual inspection of 100 randomly selected voxels that had been assigned as high quality; all of these voxels had negligible skeletal muscle contamination (PCr) or other artefacts. In healthy volunteers, there was an average PCr/ATP ratio of 0.24 ± 0.04 in liver. As PCr/ATP in skeletal muscle is approximately four, this suggests that skeletal muscle contamination contributes at most 6% of the ATP signal in our “liver” spectra, which is below the noise level in our data.

A single voxel from the CSI grid has a 50% PSF volume of 47.1 mL. Combining the high quality voxels gives an average volume of 0.77 ± 0.17 L. For fully localized 3D‐ISIS, voxel sizes have ranged between 75 and 500 mL 11, 23, 50, 51. 3D‐CSI, therefore, allows finer spatial resolution across a larger proportion of the liver than previous 3D‐ISIS studies, while retaining a feasible scan time. In this study, the finer spatial resolution is used to discard low quality regions and get a more accurate mean value for each subject, under the assumption that the tissue is homogenous in the healthy liver and in diffuse liver disease. The resolution could also be used to investigate heterogeneity in the liver, which is especially useful in focal liver diseases 52.

The mean γ‐ATP CRLB in this study was 1.6%. The literature values in the liver at 7T using a 10‐cm loop coil are 10.3% for a 20 min 3D‐CSI scan and 7.6% for a 3 min 42 s 3D‐ISIS scan 23. Accounting for the differences in acquisition time by multiplying by the square root of time, our protocol gives an 82% improvement over the 10‐cm loop 3D‐CSI scan 23, and a 43.9% improvement over the 10‐cm loop 3D‐ISIS scan 23. If the difference in volume is also accounted for, the improvements are 70% and 67%, respectively. This can be partly attributed to the difference in postprocessing. The linewidth‐constrained fit in the open‐source implementation of our fitting tool in Matlab improves the CRLB by including a relationship between the linewidths into the fitting 35. This is particularly important in the PDE/PME region of the spectra, where there are multiple overlapping peaks. Rerunning the analysis using the same prior knowledge as Chmelík et al 23 reduced the number of high quality voxels to 25 ± 10. However, the γ‐ATP CRLB of the remaining voxels was 1.65%, i.e., an improvement of 65% over the 10‐cm loop CSI scan, and 62% over the ISIS scan. The remaining difference is likely due to increased SNR due to the receive array.

The mean γ‐ATP linewidth was 34 ± 11 Hz for the Chmelík et al CSI protocol 23, compared with 48 ± 13 Hz in our protocol. Chmelík et al scanned the subject in a lateral position, and using a double‐tuned coil 23. The ^1^H loop was replaced by the ^31^P array during our protocol, and so subjects were scanned while supine to ease this coil‐swap. Both swapping the coil and scanning supine likely led to the increased linewidths in our protocol (*P* < 0.05). However, the shimming was good enough to allow consistent quantitation of individual PDE peaks.

The average voxel overlap factor of 2.5 was not accounted for when calculating the intrasubject mean and SD. This led to an artificial reduction in the intrasubject SDs given in Tables 3 and 4. Any comparison between the SDs should take this into account.

There were no significant differences between repeated scans. The Bland‐Altman plot in Figure 4 shows that there is little variation in most of the metabolite concentrations. It is possible that the small (insignificant) variation observed reflects natural variations in liver metabolite concentrations. Metabolite concentrations in the liver are known to change with nutritional state 53. And although the subjects were scanned after an overnight fast, which should increase the consistency of the results 54, no studies have been reported measuring the consistency of metabolite concentrations in the fasted liver over a long period. The 3 voxel coefficient of repeatability for the ratio of PCr/ATP in the heart at 3T was 1.1/2.14 = 51.4% 30. In comparison, the average liver α‐ATP CoR for three voxels randomly selected from the high quality voxels is 30.4%. The coefficient of repeatability is improved by the inclusion of additional voxels, as would be expected.

### Metabolite Quantitation at 7T

Our reference concentration of 2.65 mmol/L wet tissue for γ‐ATP was chosen based on an ex vivo study 39 that has commonly been used as a reference 55. However, published “absolute quantitation” γ‐ATP concentrations have varied widely (1.6 ± 0.3 to 3.8 ± 0.3 mmol/L wet tissue) 21, 47, 51, 56. Any error in the 2.65 mmol/L wet tissue concentration reference will necessarily affect direct comparison with studies performed by absolute concentration methods.

To calculate concentrations using an exogenous reference, knowledge of the $B_{1}^{-}$ fields are needed to convert the uniform noise combination of WSVD to a uniform sensitivity combination 34, 57. The Biot‐Savart method calculates the B_1_ at 0 Hz, and could, therefore, be used for the required per‐channel $B_{1}^{-}$ fields as well as to calculate flip angles. However, the combined transmit and receive magnifies any small errors and so more accurate field maps are required. These were not feasible to acquire in this study. Coil loading must also be accounted for. In this study, we used the signal from a reference fiducial. Alternatively, it would also be possible to use the ERETIC (electronic reference to access in vivo concentrations) method, which uses an electronic reference signal transmitted by a broadband antenna as the calibration reference 58. This is affected by coil loading in the same way as the NMR signal from the body. The advantage of ERETIC is that the signal is already present within the main acquisition, whereas the fiducial method requires running a series of inversion‐recovery FIDs.

The variation between centers using exogenous references is greater than the variation within studies at a particular center. It might be possible to resolve these differences in a large multicenter study, but until then each center must be self‐referenced (i.e., compare with its own normal values). In that case, relative differences can be detected in the diseased state even when variation between centers exists.

### PDE Quantitation in Healthy Volunteers

Despite the feasibility of resolving individual PME and PDE peaks at 3T and below using ^1^H‐decoupling, there has been only one study that has successfully quantified their concentrations separately 21. In other studies either PC/PE or GPC/GPE were not well resolved 51, or only ratios were reported 11, 16, 22.

The study that achieved quantification used ^1^H‐decoupling, NOE‐enhancement, and phospholipid saturation 21. Li et al did not quantify UDPG. The spectra were localized using fully sampled 3D CSI with nominal voxel size of 27 or 64 mL, allowing the quantitation of only a single voxel per subject. They reported significantly larger concentrations for all peaks except PtdC/PEP and PC (α = 0.05). However, if their results are scaled to give a γ‐ATP concentration of 2.65 mmol/L wet tissue only the GPE concentration is significantly larger in their study (*P* = 0.03), but the NAD^+^ and PC concentrations are significantly smaller (*P* < 0.0001).

At 7T, ^1^H‐decoupling, NOE‐enhancement and phospholipid saturation is unnecessary to resolve the PDE region, and the added SNR allows better localization with three to ten times smaller voxels than used by Li et al 21.

Although PC and PE are the dominant peaks in the PME region, it is likely that there are other metabolites in that region 59. It is, therefore, possible that using ^1^H‐decoupling would further enhance quantitation in that region, as well as increasing SNR. However, ^1^H‐decoupling demands high power, and might be limited by safety requirements 60. NOE enhancement is less power intense, and would increase SNR, albeit with the potential of adding variability in metabolite quantitation 60. The use of NOE in this study was prevented by hardware limitations.

**Figure 6:**
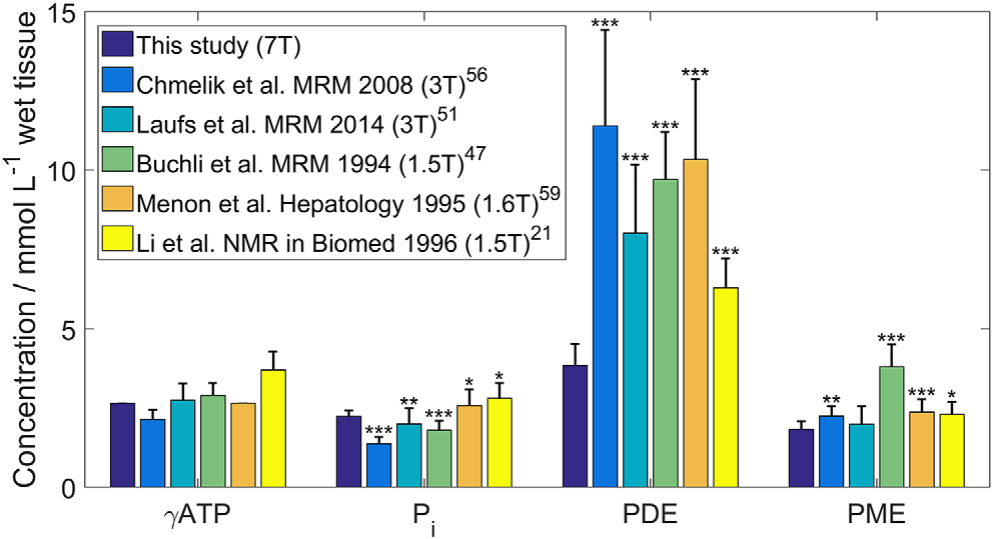
Comparison of normal liver ^31^P metabolite concentrations from this study against the literature 21, 47, 51, 56, 59. SDs are shown by the error bars on each bar. Stars indicate level of significance of the difference from this study: *P < 0.05, **P < 0.01, ***P < 0.001.

By combining the PME and PDE peaks, we can compare our results with more studies. Figure 6 compares the results of this study with five previous studies, one using an endogenous reference 59, and four using an exogenous reference 21, 47, 51, 56. Two studies ran at 3T, one at 1.6T, and two at 1.5T.

The concentration of P_i_ is significantly larger here than in refs 47, 51, 56 (*P* < 0.01; Welch's t‐test), but significantly smaller than in refs 21, 59 (*P* < 0.05). PME is significantly smaller than in refs. 21, 47, 56, 59 (*P* < 0.05), but the difference for PDE is more significant in all cases (*P* < 0.0001, 2.5–7.6 mmol/L wet tissue difference).

At low field strength, it is difficult to distinguish the PEP/PtdC peak from the PDE peak because the peaks strongly overlap 18. But the differences are still significant in the studies where the peaks were quantified separately 18, 42.

One possible explanation for this difference is the underlying resonance from endoplasmic reticulum which lies in the same spectral region as PDE. In a direct comparison between 1.5T and 3T, there was a small reduction in the underlying broad peak and a small positive difference between PME/PDE ratios 22. This effect is likely to have become more prominent at 7T, because the endoplasmic reticulum peak will have broadened even further, and may be below the SNR threshold for detection.

In vitro concentrations for GPE (2.59 ± 0.39 mmol/L wet tissue) and GPC (2.48 ± 0.48 mmol/L wet tissue) 61, converted from the published mmol/kg wet weight values using 1.054 kg/L specific gravity 40, match our concentrations more closely than the five in vivo studies we have compared our results with. On the other hand, the PC and PE concentrations measured in the in vitro study were much lower (0.17 ± 0.03 and 0.17 ± 0.04 mmol/L wet tissue) than ours, possibly due to metabolite fluctuations in cold storage which do not affect the PDE peaks 62, but it is also likely that we are including the signal from smaller peaks in the region 61.

### Patient Data

The spatial coverage in the patient group was not significantly different than in healthy volunteers. However, as would be expected, patients with very high BMI (e.g., the patient with BMI of 43.9 kg.m^‐2^) allow much lower coverage than the others because the liver is further away from the RF coil. An average γ‐ATP linewidth of 43 ± 11 Hz in the patient group indicates that the shim was also not significantly different from in healthy volunteers. Only NAD^+^ has a significantly larger variance than in healthy volunteers (F‐test; *P* = 0.003), and the residual PCr signal is not significantly higher. Our protocol is, therefore, still effective in patients with cirrhotic livers.

While the concentration of γ‐ATP is not expected to change much within a normal population, this might not necessarily be true in patients 63. Therefore, all comparisons of volunteer and patient concentrations should keep the possible differences in γ‐ATP concentration between the groups in mind. One reason for a change in ATP concentration might be the presence of fatty infiltrations. This could be addressed in future studies by a lipid partial volume correction.

We saw a significant 12% reduction in P_i_ (*P* = 0.005), which agrees with the 18% change reported by Dezortova et al in patients with cirrhosis 14. A reduction in P_i_ has been correlated with an increase in hepatic inflammation 64, 65, 66. Dezortova et al also found a significant 34% reduction in PDE, whereas we saw a significant 21% increase in GPE (*P* = 0.04). This difference is partially accounted for by the 23% reduction in PEP/PtdC (*P* = 0.02). Accounting for the effect of Dezortova et al's reported 20% reduction in ATP on the quantification would account for some of this difference, but would also give a significant reduction in the PME concentration, which was not reported by Dezortova et al. The remaining change in PDE, and the increase in PME might be explained by a variation in the underlying phospholipid signal.

## CONCLUSIONS

We report for the first time T_1_ values for PtdC, NAD^+^, and UDPG in human liver at 7T. We used these values to determine a full set of metabolite concentrations in two groups: ten volunteers and eleven patients with cirrhosis. Scanning at 7T enables resolution of the PDE region, without the need for ^1^H‐decoupling. Using a 16‐element receive array gives high quality spectra from voxels across the liver. We report comparable quality of data in patients with cirrhosis and in healthy volunteers. 7T ^31^P spectroscopy with a receive array is a powerful tool for studying metabolism in the diseased liver.

## Supporting information

Additional Supporting Information may be found in the online version of this article

**Table S1**. List of Bloch simulation fitting steps for T_1_ analysis.
**Table S2**. List of prior knowledge used for fitting the standard protocol. The phases of all peaks were additionally constrained to be the same as all the other peaks.
**Table S3**. Mean ± SD of ratios to total phosphate for 10 normal volunteers and 11 patients with cirrhotic livers from a 28min 3D CSI acquisition. Total phosphate was calculated as the sum of all visible peaks. The normal liver ratios are from the first volunteer scan. Significant differences between healthy and cirrhotic ratios are marked with stars: * *P* < 0.05, ** *P* < 0.01.
**Fig. S1**. The blue line shows the peak $B_{1}^{+}$ (i.e., with the coil at maximum voltage) for the 28 × 27 cm^2^ transmit loop calculated using the Biot‐Savart law. The red x denote direct measurements of B_1_ in the cube by repeated acquisition of FIDs with 4 ms excitation, 1500 ms T_R_, 4 preparation scans and 4 averages followed by fitting using the phantom T_1_ previously determined in fully relaxed inversion recovery experiments. The Biot‐Savart calculated $B_{1}^{+}$ values are accurate to within 10%. This leads to < 0.5% error in saturation correction on a single peak, and <1% error on the reported concentrations.
