# Supplementary material for: Phosphodiester content measured in human liver by in vivo 31P MR spectroscopy at 7 tesla
Source: Magn Reson Med. 2017 Feb 28;78(6):2095–105. doi: 10.1002/mrm.26635 (PMC5697655; doi:10.1002/mrm.26635)
Supplement: Supplementary file 1 — Table S1. List of Bloch simulation fitting steps for T1 analysis. Table S2. List of prior knowledge used for fitting the standard protocol. The phases of all peaks were additionally constrained to be the same as all the other peaks. Table S3. Mean ± SD of ratios to total phosphate for 10 normal volunteers and 11 patients with cirrhotic livers from a 28min 3D CSI acquisition. Total phosphate was calculated as the sum of all visible peaks. The normal liver ratios are from the first volunteer scan. Significant differences between healthy and cirrhotic ratios are marked with stars: * P < 0.05, ** P < 0.01. Fig. S1. The blue line shows the peak B1+ (i.e., with the coil at maximum voltage) for the 28 × 27 cm2 transmit loop calculated using the Biot‐Savart law. The red x denote direct measurements of B1 in the cube by repeated acquisition of FIDs with 4 ms excitation, 1500 ms TR, 4 preparation scans and 4 averages followed by fitting using the phantom T1 previously determined in fully relaxed inversion recovery experiments. The Biot‐Savart calculated B1+ values are accurate to within 10%. This leads to < 0.5% error in saturation correction on a single peak, and <1% error on the reported concentrations. [file MRM-78-2095-s001.docx]

# Supporting Information

# T_1_ acquisition and analysis

A 449V, 29.696 ms HS8_­_ inversion pulse with time-bandwidth product R = 24 was used. This was followed by a 449V excitation and readout at an inversion time of 50 ms and at 20 x 500ms intervals thereafter. After a 5.2s interval, there were three further excitations and readouts. The signals were localized using a 6 × 6 × 4 CSI grid interpolated to 8 x 8 x 8 over a 200 × 240 × 270 mm^3^ FOV. The LL-CSI sequence was run twice in each subject, first with inversion at +666Hz (relative to skeletal phosphocreatine (PCr)) and second at -584Hz (relative to PCr). Each acquisition took 34 min 12s. A BISTRO saturation band was placed over the skeletal muscle at the maximum voltage possible within SAR limits (1).

For each voxel manually chosen for analysis, frequency-domain spectra were simulated for every TI and both inversions.

The exact RF pulse waveforms and RF and ADC event timings for both inversion and excitation were used. Perfect spoiling after inversion and readout was assumed. A T_2_^*’^ term due to B_0_ inhomogeneity was combined with individual T_2_s to give independent T_2_^*^ terms for each peak.

A Bloch simulation for each peak was separately performed in the time-domain and the results were summed. Multiplets were modelled with separate peaks with relative amplitude and frequency offset. The resultant free induction decays were Fourier transformed to the frequency-domain and zero-order phase was applied.

These model spectra were fitted to the experimental spectra using the Matlab function ‘lsqcurvefit’. Both model and experimental spectra were apodized to stabilize the fit. Bounds were applied to avoid unphysical fit variables, e.g. negative relaxation times and M_0_.

There were several fittings steps, with specific TIs and inversions fitted with certain variables in each step. The initial values for frequency, T_2_ and zero order phase are supplied by fitting with an open-source, Matlab spectroscopy fitting tool (2). For each voxel, the initial M_0_ was manually adjusted to match the spectrum. At each step, the fitted variables were given with Cramér-Rao lower bounds (3). The final step of each fit was manually inspected to ensure high quality.

1. Luo Y, de Graaf RA, DelaBarre L, Tannus A, Garwood M. BISTRO: An outer-volume suppression method that tolerates RF field inhomogeneity. Magn Reson Med. 2001; 45(6):1095-102.

2. Purvis LAB, Clarke WT, Biasiolli L, Robson MD, Rodgers CT. Linewidth Constraints in Matlab AMARES using per-Metabolite T_2_  and per-Voxel Delta B_0_. In the Proceedings of the 22^rd^ Annual Meeting of ISMRM, Milan, Italy, 2014. p. 2885.

3. Cavassila S, Deval S, Huegen C, van Ormondt D, Graveron-Demilly D. Cramér-Rao bounds: an evaluation tool for quantitation. NMR Biomed. 2001; 14(4):278-83.

| Step | Fitting variables | MOLLI step | Inversion frequency relative to PCr |
| --- | --- | --- | --- |
| 1 | Voxel M_0_ scaling factor and all peak T_2_ | Fully relaxed | +666Hz |
| 2 | All peaks M_0_, acquisition phase. | Fully relaxed | +666Hz |
| 3 | RF scaling factor | All acquisitions | +666 and -584 Hz |
| 4 | All peaks M_0_ and T_2_ | Fully relaxed | +666Hz |
| 5 | All peak T­_1_ and M_0_ | All acquisitions | +666 and -584 Hz |
| 6 | All peak parameters, RF scaling factor, and global zero order phase | All acquisitions | +666 and -584 Hz |

**Supporting Table S1:** List of Bloch simulation fitting steps for T_1_ analysis

# Prior knowledge used in main protocol

|  | **β-ATP** | **α-ATP** | **γ-ATP** | **PCr** | **P_i_** | **GPC** | **GPE** | **PC** | **PE** | **NAD^+^** | **UDPG** | **PEP/PtdC** |
| --- | --- | --- | --- | --- | --- | --- | --- | --- | --- | --- | --- | --- |
| **Multiplet** | | | | | | | | | | | | |
| Ratio | 1:2:1 | 1:1 | 1:1 | - | - | - | - | - | - | - | - | - |
| Splitting (Hz) | 15 | 16 | 15 | - | - | - | - | - | - | - | - | - |
| **Additional linewidth** | - | 0.93 | 0 | - | 16.08 | 5.30 | -10.30 | 0.78 | -9.56 | 37.25 | 47.18 | 41.08 |
| **Initial Values** **(Bounds)** | | | | | | | | | | | | |
| Chemical shift (ppm) | -16.00 (-inf ,-10) | -7.40 (-inf, inf) | -2.21  (-inf, -0.5) | 0.00  (-0.5, 0.5) | 5.37  (0, 10) | 3.19  (3,6) | 3.71  (3, 6) | 6.70 (6,8) | 7.30 (6.3, 9) | -8.25 (-15,-5) | -9.48 (-15,-5) | 2.20 (2,5) |
| Linewidth (Hz) | 40 (0, 200) | 40 (0, inf) | 30 (0, inf) | 5 (0, 50) | 50 (20,100) | 20 (0, inf) | 20 (0, inf) | 10 (0, inf) | 10 (0, inf) | 20 (0, inf) | 20 (0, inf) | 20 (0, inf) |
| Amplitude | 3 (0, inf) | 3 (0, inf) | 3 (0, inf) | 0.5 (0, inf) | 2 (0, inf) | 2.5 (0, inf) | 2.5 (0, inf) | 1 (0, inf) | 1 (0, inf) | 0.5 (0, inf) | 0.6 (0, inf) | 0.6 (0, inf) |
| Phase (degrees) | 0 (0, 360) | 0 (0, 360) | 0 (0, 360) | 0 (0, 360) | 0 (0, 360) | 0 (0, 360) | 0 (0, 360) | 0 (0, 360) | 0 (0, 360) | 0 (0, 360) | 0 (0, 360) | 0 (0, 360) |

**Supporting Table S2:** List of prior knowledge used for fitting the standard protocol. The phases of all peaks were additionally constrained to be the same as all the other peaks.

# Flip angle validation


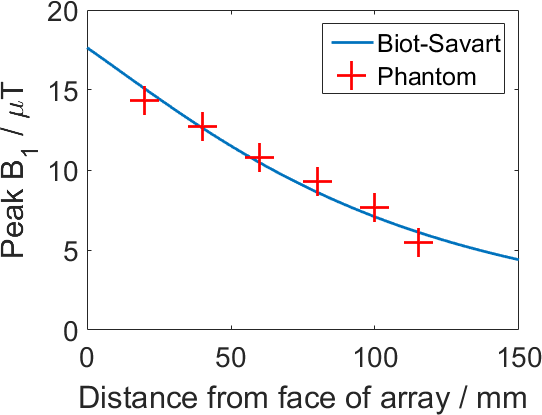


**Supporting Figure S1:**  The blue line shows the peak B_1_^+^ (i.e. with the coil at maximum voltage) for the 28 × 27 cm^2^ transmit loop calculated using the Biot-Savart law. The red x denote direct measurements of B_1_ in the cube by repeated acquisition of FIDs with 4ms excitation, 1500ms T_R_, 4 preparation scans and 4 averages followed by fitting using the phantom T_1_ previously determined in fully relaxed inversion recovery experiments. The Biot-Savart calculated B_1_^+^ values are accurate to within 10%. This leads to <0.5% error in saturation correction on a single peak, and <1% error on the reported concentrations.

# Ratios to total phosphate

| **Metabolite** | **Normal liver ratio to total phosphate / %** | **Cirrhotic liver ratio to total phosphate / %** |
| --- | --- | --- |
| α-ATP | 14.1 ± 0.9 | 14.4 ± 0.8 |
| γ-ATP | 13.7 ± 0.5 | 13.6 ± 0.5 |
| P_i_ | 11.3 ± 1.0 | 10.0 ± 1.2** |
| GPC | 11.7 ± 2.0 | 12.8 ± 2.8 |
| GPE | 7.6 ± 1.2 | 9.1 ± 2.2 * |
| PC | 5.3 ± 0.8 | 4.7 ± 1.2 |
| PE | 3.8 ± 0.7 | 3.8 ± 0.6 |
| PtdC/PEP | 6.8 ± 1.3 | 5.4 ± 1.6 * |
| NAD^+^ | 12.2 ± 0.5 | 12.2 ± 1.7 |
| UDPG | 10.1 ± 1.0 | 10.0 ± 1.6 |

**Supporting Table S3:** Mean ± SD of ratios to total phosphate for 10 normal volunteers and 11 patients with cirrhotic livers from a 28min 3D CSI acquisition. Total phosphate was calculated as the sum of all visible peaks. The normal liver ratios are from the first volunteer scan. Significant differences between healthy and cirrhotic ratios are marked with stars: * *P* < 0.05, **  *P* < 0.01.
